# Atypical periprosthetic femoral fracture associated with long-term bisphosphonate therapy

**DOI:** 10.1186/s13018-020-01941-x

**Published:** 2020-09-15

**Authors:** Dávid Dózsai, Tamás Ecseri, István Csonka, István Gárgyán, Péter Doró, Ákos Csonka

**Affiliations:** 1 Traumatology Department, University of Szeged, Semmelweis u. 6, Szeged, 6725 Hungary; 2 Clinical Pharmacy Department, University of Szeged, Szikra utca 8, Szeged, 6725 Hungary

**Keywords:** Periprosthetic fracture, Atypical femoral fracture, Bisphosphonate, Atypical periprosthetic femoral fracture

## Abstract

**Background:**

Atypical femoral fracture is one of the many complications after the long-term use of bisphosphonates. The American Society for Bone and Mineral Research has officially excluded periprosthetic femoral fractures (PFFs) from the definition of atypical femoral fractures (AFFs). Several case reports found that PFFs can occur with characteristics similar to those of AFFs. The purpose of our study was to evaluate the proportion of atypical fractures among Vancouver type B1 fractures, and to determine the association between the long-term use of bisphosphonates and the occurrence of atypical periprosthetic femoral fractures (APFFs).

**Methods:**

In this retrospective study, we reviewed 41 patients with Vancouver type B1 periprosthetic fractures between January 1, 2011 and December 31, 2018. We classified them into two groups, namely atypical and typical PFFs, based on the fracture morphology. We noted the proportion of atypical periprosthetic fractures among B1 fractures and identified risk factors.

**Results:**

Among the 41 PFFs, 5 (13%) fractures were classified as atypical PFF based on the radiological characteristics. The longer duration of bisphosphonate use was probably the only independent risk factor that significantly increases the occurrence of APFF (*p* = 0.03, 0.08 (CI 0.008 – 0.16)). There were no significant differences in age, gender, body mass index, comorbidities, corticosteroid use, positioning of the femoral stem, the method of fixation (cemented or cementless) and time lapse from before the primary prosthesis implantation to the PFF in the development of atypical fracture type.

**Conclusions:**

There seems to be a correlation between the long-term intake of bisphosphonates and the atypical periprosthetic fracture. Atypical femoral fracture can also occur in the periprosthetic form.

**Trial registration:**

Study number: 22/2019-SZTE, http://www.klinikaikutatas.hu/hu/kutatasetika/jovahagyott-vizsgalatok-koezerdeku-adatai/category/25-jovahagyott-vizsgalatok-kozerdeku-adatai-rkeb-2019.html?download=985:22-2019.

## Introduction

Today, osteoporosis is a disease that affects many millions of people worldwide. About 7-10% of the Hungarian population suffer from it [1]. The use of bisphosphonates has significantly reduced the risk of osteoporotic fractures and hence it has reduced the burden of health care costs [2].

**Fig. 1:**
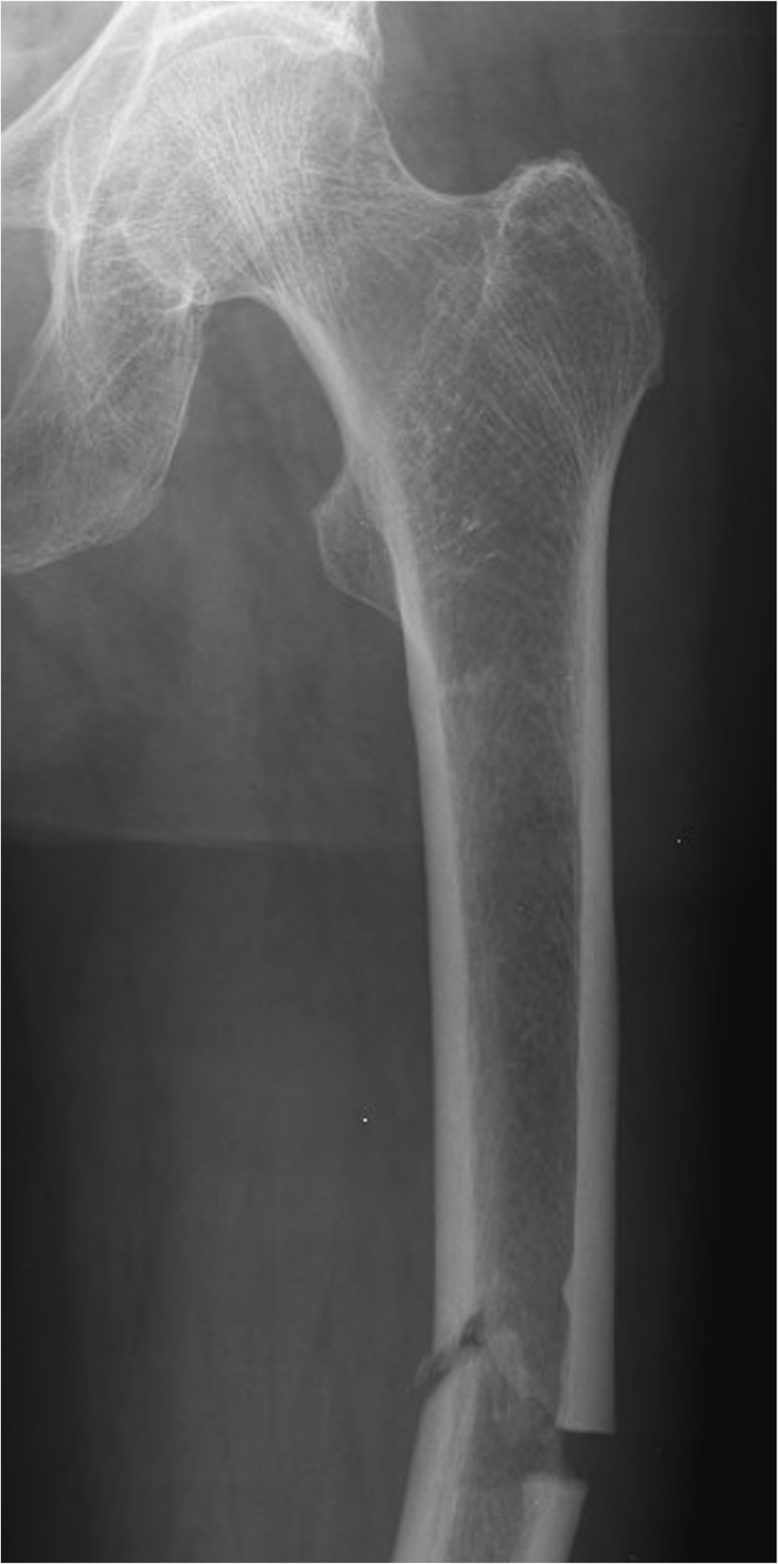
A 68-year-old patient with an atypical femoral fracture after a low energy fall

**Fig. 2:**
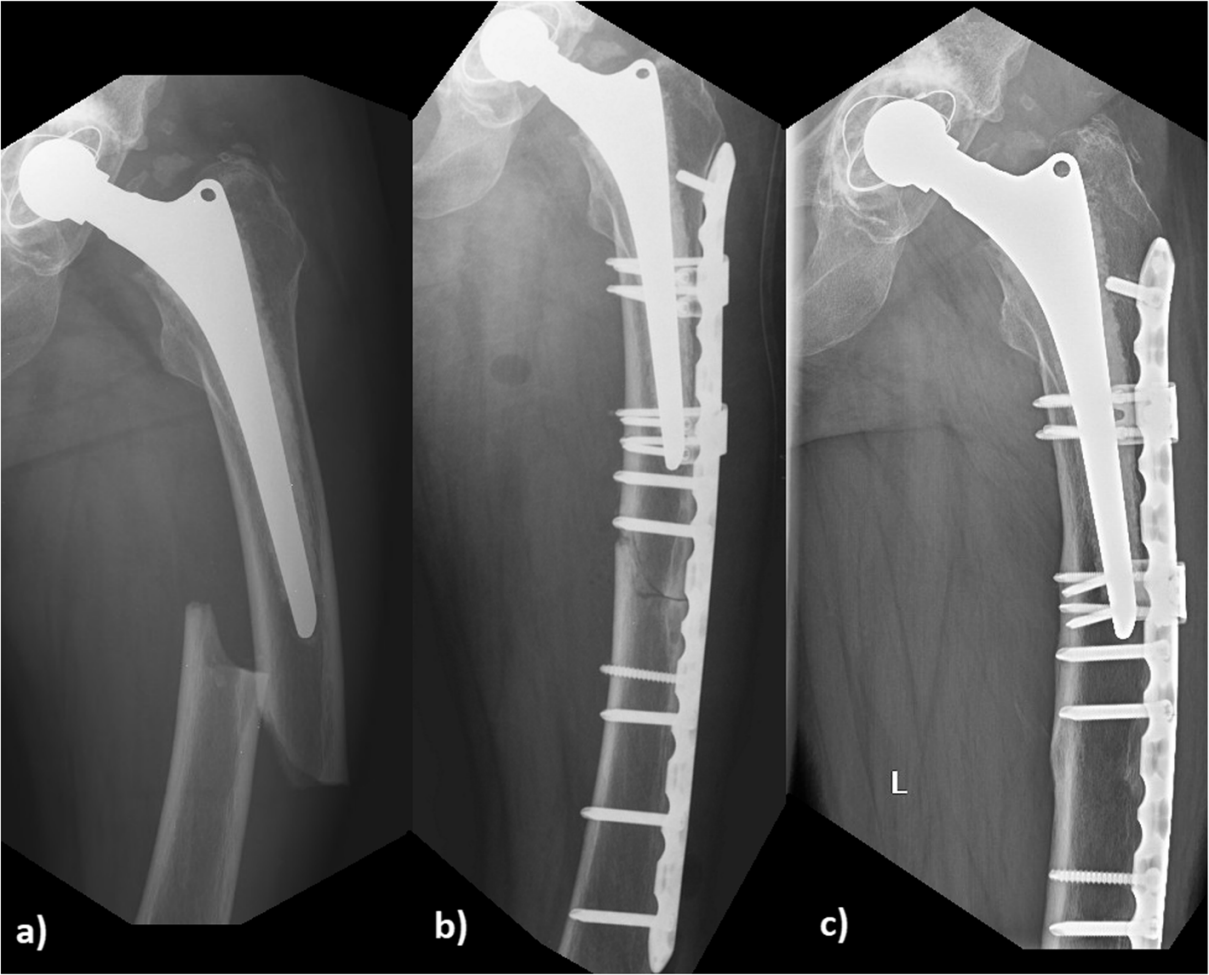
**a** 76-year-old female patient with an atypical periprosthetic femoral fracture, who took bisphofonates for 11 years. **b** Postoperative x-ray of the atypical fracture (operative fixation method with an 18 hole locking compression plate and 2 pieces of attachment). **c** X-ray of a healed atypical fracture after 1 year

Bisphosphonates have been known to have beneficial effects on the skeletal system such as the reduced risk of fracture. They inhibit resorption of the bone by blocking the action of osteoclasts, so the development of fractures is decreased by minimizing bone loss [3, 4]. Microfractures, forming in the bone, are decreasing indirectly; but the replacement of previously developed microfractures with a new bone matrix is also impaired. This has led to the appearance of atypical femoral fractures (AFF), as a new type of fracture, caused by an artificially developed low bone turnover due to the long-term intake of bisphosphonates [5]. Atypical femoral fracture (AFF) is a stress fracture occurring with low energy or no trauma and it has a characteristic radiographic appearance (Fig. 1) [6]. With atypical subtrochanteric and middle third femoral fractures, periprosthetic fractures should be treated as exclusion criteria based on the recommendation of the American Society for Bone and Mineral Research, published in 2014 [6]. Over the past few years, several case reports have stated that periprosthetic femoral fractures (PFFs) can occur with similar features to those of atypical femoral fractures (AFFs) arising from the long-term use of bisphosphonates (Fig. 2) [7–11]. Atypical periprosthetic femoral fractures (APFFs) are prevalent in the older generation because it is common to have joint implants in this population, they more likely to suffer from osteoporosis, and to have other comorbidities (e.g., diabetes, vitamin D deficiency and the use of the proton pump inhibitor) in their medical record [12]. To the best of our knowledge, the main principal cause, etiology, diagnostic criteria and therapeutic recommendations of APFFs still have not been clearly defined [10].

In our study, we examined one of the possible side-effects of the long-term intake of bisphosphonates on the skeletal system and the incidence of atypical periprosthetic femoral fractures. We analyzed the prevalence of atypical periprosthetic fractures falling into the class of Vancouver type B1 fractures, and we looked for a correlation between the long-term use of bisphosphonate and APFF.

## Methods

We carried out a retrospective study between 1st January, 2011 and 31st December, 2018 at the Traumatology Department of University of Szeged. We reviewed the medical records and radiographs of 109 patients with hip replacements who had periprosthetic fractures afterwards. Inclusion criteria were patients who were over 50 years and had had a low energy trauma.

Out of 109 patients, 15 patients who were polytraumatized and 10 patients with incomplete medical records were excluded. After a radiologic review, we excluded patients who had Vancouver type A (*n* = 2), B2 (*n* = 12), B3 (*n* = 4) or C (*n* = 25) fractures. The remaining 41 patients (Vancouver type B1 periprosthetic fracture) were subjects of our study (see Table 1).

**Table 1:**
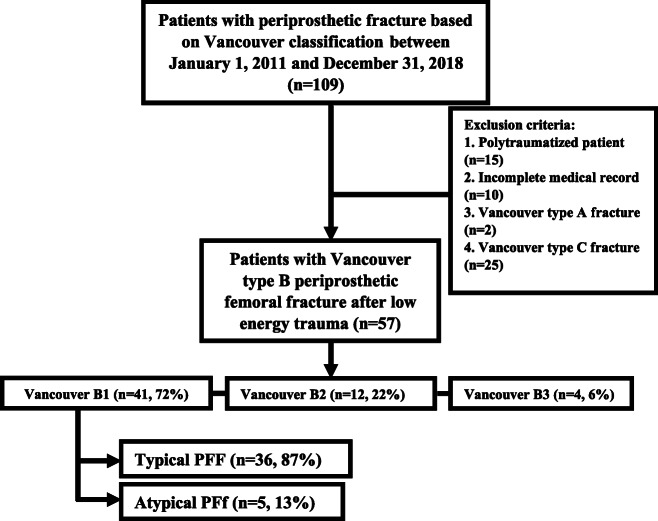
Flow diagram of the retrospective study

First of all, we took into account the age, gender, body mass index (BMI), comorbodities (e.g., hypertension, diabetes, rheumatoid arthritis, thyroid disease, neurologic disease, malignancy, osteoporosis) and the use of bisphosphonate and glucocorticoid. We evaluated the position of the femoral stem on the radiographs and the method of fixation (cemented or cementless stem). Then we determined the interval between PFF and prior hip arthroplasty and the time of bone formation. In every case the bisphosphonate therapy applied was alendronate.

In order to establish the diagnosis of atypical femoral fractures, we used the revised criteria of the American Bone and Mineral Research Taskforce (see Table 2) [6]. A diagnosis of atypical femoral fractures was made when four or more of the major criteria were met.

**Table 2:** ASBMR Task Force 2013 Revised Case Definition of AFFs [6]

Major criteria	Minor criteria	Exclusion criteria
• minimal or no trauma • Transverse or slightly oblique fracture line • Complete fractures extend through both cortices and may be associated with a medial spike; incomplete fractures involve only the lateral cortex • non or minimally comminuted fracture • Localized periosteal or endosteal thickening of the lateral cortex is present at the fracture site	• increase in cortical thickness of the femoral diaphyses • Unilateral or bilateral prodromal symptoms such as dull or aching pain in the groin or thigh • Bilateral incomplete or complete diaphysis fractures • Delayed healing	• femoral neck fracture • intertrochanteric fractures with spiral subtrochanteric extension • periprosthetic fractures • pathological fractures related to primary or metastatic bone tumors and miscellaneous bone diseases

We applied locking compression plates (LCP) in each case to fixate the atypical periprosthetic fractures, completing it with cerclage, cable and attachment, where necessary (Fig. 2.).

### Statistics

Univariate statistical comparisons were performed using the Mann-Whitney U test for continuous variables, and Fisherˊs Exact test for categorized data. The confidence level was set at 95%. Then, a multivariable regression analysis was performed for 7 risk factors that displayed the most significant value based on a univariate comparison. Also the chosen risk factors for the multivariable regression analysis were the most common in the literature as main risk factors of AFF (eg. RA, BP use, gender (female), diabetes). All the analyses were conducted using Windows Microsoft Excel (2016 version).

## Results

Among 41 Vancouver type B1 PFFs, 5 (13%) were classified as atypical PFF. The mean age of patients with typical PFFs was 79.5 (54-94) years, compared to 80 (76-85) years for patients with atypical PFFs. As regards gender, there were 27 men and 14 women. Atypical fractures occurred only in women (*n* = 5). There were no significant differences in age, gender, BMI, comorbodities, the proportion of osteoporosis (T score < − 2.5), positioning of the femoral stem and the method of fixation between the typical PFF and atypical PFF group. In terms of gender, atypical fractures occurred only in women, which presumably did not play any significant role in the development of this fracture type (*p* = 0.26). There was no significant difference between the two groups (*p* = 0.27) regarding the length of time of bone development, but the upper leg bone took longer to heal in the atypical group. We observed a significant correlation between the history of bisphosphonate use (*p* = 0.01) and the duration of bisphosphonate therapy (p = 0.01) in the development of AFF in our univariate analysis.

The only independent significant risk factor was probably the duration of bisphosphonate use (*p* = 0.03, 0.08 (CI 0.008 – 0.16)) in APFF in terms of age, gender, history of rheumatoid arthritis and osteoporosis, the duration and history of bisphosphonate use and the time lapse from before the primary prosthesis implantation to the PFF in the multivariate regression analysis. In our regression model we got an R squared value of 0.43 (see Table 3).

**Table 3:** Demographics of typical PFF group and atypical PFF group

Vancouver type B1 fracture distribution	Typical PFF (***n*** = 36, 87%)	%	Atypical PFF (n = 5, 13%)	%	***p***-value
Mean age	79.5 (54-94)		80 (67-85)		0.24
Gender (male/female)	9/27		0/5		0.26
BMI – body mass index	24.9 ± 3.3		23.2 ± 1.8		0.53
**Chronic diseases**					
Diabetes	7	19.44%	0	0%	0.37
Rheumatoid arthritis	1	2.78%	1	20%	0.21
Hypertension	35	97.22%	5	100.0%	0.87
Thyroid disease	5	13.89%	2	40%	0.66
Malignancy	3	8.33%	0	0%	0.62
Neurologic disease	9	25.00%	2	40%	0.29
Osteoporosis	16	44.44%	4	80%	0.20
History of bisphosphonate use	5	13.88%	4	80%	0.01
Duration of bisphosphonate use (years average)	4 ± 0.7		8.2 ± 5.5		0.01
Corticosteroid use	1	2.78%	0	0%	0.87
**Stem position**					
Central	32	88.89%	4	80%	0.39
Varus	2	2.78%	1	20%	0.29
Valgus	2	2.78%	0	0%	0.76
Cemented stem	31	86.11%	5	100%	0.50
Cementless stem	5	13.89%	0	0%	0.50
Duration of healing process (months average)	5.7 ± 1.4		9.2 ± 4.7		0.27
Interval between prior arthroplasty and PFF (years average)	10 ± 8		6 ± 3.5		0.26

## Discussion

Atypical femoral fractures typically occur as stress fractures. Prodomal pain in the thigh preceding the fracture was described previously in the literature. Radiological characteristics are usually bilateral, including microfracture and the lateral cortex thickening [6]. Radiography should include bilateral views of the full length of each femur in the case of atypical femoral fractures arising from lateral cortex thickening and microfracture, which might suggest impending fractures. It is known that the frequency of late periprosthetic fracture is the highest in the case of fixation of the cemented stem. However, periprosthetic fracture usually occurs within six months in cementless hip arthroplasty, but it is not correlated with the atypical fracture type [13]. Atypical fractures occurred in cemented hip arthroplasty, but they did not play any significant role in the development of an atypical fracture type (*p* = 0.50). In terms of positioning of the stem, it is known that varus positioning increases the stress and fractures are more likely to occur at the tip of the femoral stem [14].

None of the positions (central, varus, valgus) played a significant role in the development of atypical fractures related to the location of the prosthetic stem. The surgical treatment of the periprosthetic Vancouver type B1 fractures is based on the LCP systems, and these can be combined with cerclage, cable or plate attachment, and moreover, allograft can be implanted in order to stabilize the fixation of the fracture in the case of bone loss [15, 16].

In our study no significant difference was found between the atypical periprosthetic B1 fractures and the typical periprosthetic fractures in terms of fixation, but it is should be recalled that a delayed healing process, higher mortality rate and more complications can occur after osteosynthesis [6, 17].

PFFs represent a big challenge for orthopedic surgeons because the observed frequency of these fractures due to the rising number of patients with prosthetics is increasing [15]. The American Society for Bone and Mineral Research excluded periprosthetic fractures in the case of atypical fracture, but they were mainly published as case studies in the literature [7–9, 18].

In this study among the 41 PPFs, 5 fractures were classified as atypical PFF based on the radiological characteristics and among these, 4 were associated with long-term bisphosphonate use. This retrospective study has some limitations, such as the small patient number, incomplete patient history (no knowledge of prodromal pain in the thigh) and the lack of radiographs of the contralateral femur. By case definition according to the second, revised recommendation of the American Society for Bone and Mineral Research no association was found between atypical fractures and other comorbodities or the use of drugs such as bisphosphonates. Case studies report a significant association between AFFs and bisphosphonate use; even though the strength of the association and size of the effect vary, just the relative risk of patients with AFFs taking BPs is high [6, 19]. However, long-term use may be associated with AFFs [6]. Although the use of bisphosphonate was excluded in the case definitions of AFF, their long-term use is still considered as one of the most important pathophysiologic mechanisms of AFF. As reported in the literature, there is a strong association between atypical femoral fracture and bisphosphonate use especially in the case of long-term treatment (> 5 years) [17, 20].

A strong correlation was found between those taking bisphosphonates and atypical fractures including periprosthetic types according to a multicentric study that processed 10 years of follow-up studies of the National Trauma Registries in Canada and the USA [10]. Level I evidence might be needed to demonstrate that bisphosphonates play a leading role in the development of atypical fractures. In the literature, studies on atypical periprosthetic fractures are mainly case reports [10, 17, 21].

In our study, the long-term use of bisphosphonate seemed to be the only independent risk factor associated with atypical periprosthetic femoral fractures. Previous related publications arrived at a similar conclusion [8–10, 12].

## Conclusions

On the basis of our results and the literature it appears that atypical femoral fractures can occur in the periprosthetic form and display a significant correlation with bisphosphonate use [9, 17].

The medical management of atypical fractures is a big challenge and the outcome is much poorer than that of the typical fractures because of the delayed healing process, poor bone consolidation, difficulty of fracture fixation and high mortality rate [22–24]. Nevertheless, our results indicate that clinicians should consider the possibility of atypical fracture, when periprosthetic Vancouver type B1 fracture occurred if long-term bisphosphonate therapy is mentioned in the patient history. Bisphosphonate therapy should be applied carefully, always bearing in mind the risk-benefit ratio. Keeping up to date with the latest antiresorptive medications and follow up-care of patients is crucial for correct patient treatment.

## Data Availability

Raw data were generated at the university hospital. Derived data supporting the findings of this study are available from the corresponding author on request.
